# Complete mitochondrial genome of white-eyed parakeet (*Psittacara leucophthalmus*): the basal species to other *Psittacara*

**DOI:** 10.1080/23802359.2016.1258344

**Published:** 2016-11-22

**Authors:** Adam Dawid Urantówka, Paweł Mackiewicz

**Affiliations:** a Department of Genetics, Wroclaw University of Environmental and Life Sciences, Wroclaw, Poland;; b Department of Genomics Faculty of Biotechnology, University of Wrocław, Wrocław, Poland

**Keywords:** Arini, mitochondrial genome, *Psittacara leucophthalmus*, Psittaciformes, white-eyed parakeet

## Abstract

Recently resurrected *Psittacara* genus is one of the 19 recognized in parrot tribe *Arini*. The status of taxa within *Psittacara* remains controversial because some forms are treated as species or subspecies depending on authorities. Evolutionary history of *Psittacara* is also unclear because related phylogenetic clades contain taxa from distant and non-overlapping regions. However, the basal placement of *Psittacara leucophthalmus* with wide South American distribution suggests that other taxa with restricted range could emerge by a local split of larger population. We sequenced *P. leucophthalmus* mitogenome to increase the set of sequences required to determine taxonomic level and phylogeny of *Psittacara* taxa.

*Arini* tribe is the most taxon-rich among neotropical parrots (*Arinae* subfamily) (Schodde et al. 2013). The majority of *Arini* genera are divided into two morphologically diverse groups, macaws and conures. Among nine conure genera (Remsen et al. 2016), three (*Eupsittula*, *Thectocercus,* and *Psittacara*) were recently resurrected after the molecular revision of the previously broadly defined *Aratinga* genus (Remsen et al. 2013).

Taxonomy of *Psittacara* genus is still controversial. Gill and Donsker (2016) recognize 11 extant *Psittacara* species. However, three of them (*brevipes*, *rubritorquis,* and *strenuus*) are treated as subspecies of *holochlorus* according to Dickinson and Remsen (2013) and Clements et al. (2016) in contrast to others (Juniper & Parr 1998; Forshaw 2010; Remsen et al. 2013) assuming that they have a species rank. Based on differences in plumage and habitat, Del Hoyo and Collar (2014) treated *frontatus* (the southern *P. wagleri* subspecies) as a separate species. Similarly, Arndt (2006) elevated the Andean *P. mitratus alticola* to the new species rank and described a new species *P. hockingi* from Peru. However, these species were not recognized by Remsen et al. (2013). A museum specimen of *hocking* (identified as *P. mitratus*) seems closely related to *P. wagleri* but its relationship with other *Psittacara* was not studied molecularly.

So far, only few phylogenies for *Psittacara* were published. Kirchman et al. (2012) using mitochondrial genes (*nd2* and *coxI*), denied conspecificity of *wagleri* and *mitratus* species, which were suggested by Collar (1997). Analyses of *nd2* by Urantowka et al. (2014a) showed that *brevipes* should be a separate species, sister to the clade of *P. finschi* and *holochlora*/*rubritorquis*. However, the taxonomic status of *rubritorquis* is still unclear, because *strenuus* and *brewsteri* (probable *holochlora* subspecies) were not included in the phylogenies.

More molecular data, especially complete mitochondrial genomes (Nabholz et al. 2013), are required to reconstruct a precise phylogeny of *Psittacara* and establish a taxonomic level of its species/subspecies. Several complete mitogenomes of *Psittacara* taxa are already available (Urantowka et al. 2014a, 2016a, 2016b). To increase this set, we sequenced the mitogenome (16,966 bp, accession number KF444466) from *P. leucophthalmus.* Although morphology of the analyzed specimen (Polish captive bird) was absolutely typical for *leucophthalmus*, we proved its taxonomic affiliation in phylogenetic analyses of *nd2* sequences including all available *Psittacara* taxa. The obtained tree (Figure 1) revealed that the analyzed individual groups significantly with two other representatives of its species. This species is basal to all *Psittacara* taxa and the next diverged lineage is *P. wagleri*. These positions are important to explain *Psittacara* diversification and migration routes, similarly to Yellow-headed Amazon parrots, for which Venezuelan *Amazona barbadensis* species played such pivotal role (Urantówka et al. 2014b). The basal placement of *P. leucophthalmus* and its wide distribution in South America suggests that other *Psittacara* species could emerge by a local split of a larger population. However, further phylogeographic history of *Psittacara* is complicated because subsequent clades contain parrots from distant and non-overlapping regions such as the central and northern part of South America, Central America, and Great Antilles (Figure 1).

**Figure 1.**
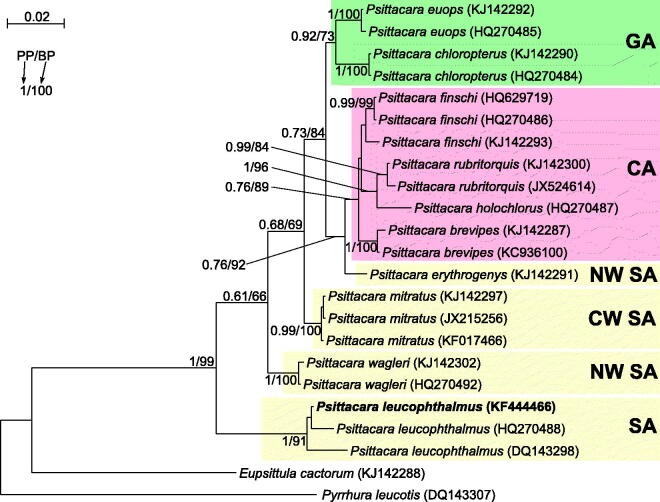
The phylogenetic tree obtained in MrBayes for *nd2* gene indicating that the studied individual (bolded) belongs to *P. leucophthalmus*. The parrot is kept in aviculture and its blood sample from which DNA was isolated is available in the laboratory at the Department of Genetics in Wroclaw University of Environmental and Life Sciences under the number PL16966. Clades with taxa inhabited different geographic regions were marked by various colours/shading: CA – Central America; GA – Greater Antilles; NW SA – northwest South America; CW SA – central-western South America; SA – a vast part of South America. Migration and colonization routes of *Psittacara* parrots are not easy to infer because related clades include taxa, which currently have distant and restricted distributions. *P. erythrogenys* is the only South American species that is placed between Central American (*finschi*, *rubritorquis*, *brevipes*, *holochlora*) and Greater Antillean (*euops*, *chloropterus*) taxa. Similarly, *P. wagleri* with the most northern distribution in South America is placed between *mitratus* from the central South America, and *leucophthalmus* widespread in the large part of the continent. The close relationship of north-western *P. erythrogenys* and the Central American parrots suggests migrations through the Isthmus of Panama. However, origin of parrots from Greater Antilles remains unsolved. Values at nodes, in the order shown, indicate posterior probabilities found in MrBayes (PP) and bootstrap percentages calculated in TreeFinder (BP). In the MrBayes (Ronquist et al. 2012) analysis, separate mixed substitution models were assumed for three codon positions with information about heterogeneity rate across sites as proposed by PartitionFinder (Lanfear et al. 2012). We applied two independent runs, each using eight Markov chains. Trees were sampled every 100 generations for 10,000,000 generations. After obtaining the convergence, trees from the last 3,938,000 generations were collected to compute the posterior consensus. In the case of TreeFinder (Jobb et al. 2004), the separate substitution models were selected for three codon positions according to Propose Model module in this program, and 1000 replicates were assumed in the bootstrap analysis. The posterior probabilities <0.5 and bootstrap percentages <50 were omitted.
